# Novel Na^+^/Ca^2+^ Exchanger Inhibitor ORM-10962 Supports Coupled Function of Funny-Current and Na^+^/Ca^2+^ Exchanger in Pacemaking of Rabbit Sinus Node Tissue

**DOI:** 10.3389/fphar.2019.01632

**Published:** 2020-01-29

**Authors:** Zsófia Kohajda, Noémi Tóth, Jozefina Szlovák, Axel Loewe, Gergő Bitay, Péter Gazdag, János Prorok, Norbert Jost, Jouko Levijoki, Piero Pollesello, Julius Gy. Papp, András Varró, Norbert Nagy

**Affiliations:** ^1^MTA-SZTE Research Group of Cardiovascular Pharmacology, Hungarian Academy of Sciences, Szeged, Hungary; ^2^Department of Pharmacology and Pharmacotherapy, Faculty of Medicine, University of Szeged, Szeged, Hungary; ^3^Institute of Biomedical Engineering, Karlsruhe Institute of Technology (KIT), Karlsruhe, Germany; ^4^Orion Pharma, Espoo, Finland

**Keywords:** Na^+^/Ca^2+^ exchanger, funny-current, ORM-10962, pacemaking, sinus-node

## Abstract

**Background and Purpose:**

The exact mechanism of spontaneous pacemaking is not fully understood. Recent results suggest tight cooperation between intracellular Ca^2+^ handling and sarcolemmal ion channels. An important player of this crosstalk is the Na^+^/Ca^2+^ exchanger (NCX), however, direct pharmacological evidence was unavailable so far because of the lack of a selective inhibitor. We investigated the role of the NCX current in pacemaking and analyzed the functional consequences of the I_f_-NCX coupling by applying the novel selective NCX inhibitor ORM-10962 on the sinus node (SAN).

**Experimental Approach:**

Currents were measured by patch-clamp, Ca^2+^-transients were monitored by fluorescent optical method in rabbit SAN cells. Action potentials (AP) were recorded from rabbit SAN tissue preparations. Mechanistic computational data were obtained using the Yaniv *et al*. SAN model.

**Key Results:**

ORM-10962 (ORM) marginally reduced the SAN pacemaking cycle length with a marked increase in the diastolic Ca^2+^ level as well as the transient amplitude. The bradycardic effect of NCX inhibition was augmented when the funny-current (I_f_) was previously inhibited and *vice versa*, the effect of I_f_ was augmented when the Ca^2+^ handling was suppressed.

**Conclusion and Implications:**

We confirmed the contribution of the NCX current to cardiac pacemaking using a novel NCX inhibitor. Our experimental and modeling data support a close cooperation between I_f_ and NCX providing an important functional consequence: these currents together establish a strong depolarization capacity providing important safety factor for stable pacemaking. Thus, after individual inhibition of I_f_ or NCX, excessive bradycardia or instability cannot be expected because each of these currents may compensate for the reduction of the other providing safe and rhythmic SAN pacemaking.

## Introduction

Computational modeling as well as experimental results established previously that the normal pacemaker function is not only regulated by the hyperpolarization-activated funny current (I_f_) (DiFrancesco, 1981) but is also regulated by the intracellular Ca^2+^ handling (Lakatta and DiFrancesco, 2009; Yaniv et al., 2013a; Yaniv et al., 2015; Sirenko et al., 2016). Lakatta and co-workers suggested that the sinus node (SAN) cells operate by a rhythmic clock-like oscillator system where the sarcoplasmic reticulum serves as a Ca^2+^-clock, which rhythmically discharges diastolic local Ca^2+^ releases (LCRs), and activates the forward (inward) Na^+^/Ca^2+^ exchanger (NCX) current to accelerate the diastolic depolarization and facilitates the membrane-clock (M-clock) (Yaniv et al., 2015). Recent experimental results further suggest that these clocks work tightly coupled since the M-clock regulates the Ca^2+^ influx and efflux while the NCX also regulates the diastolic depolarization forming a coupled-clock system. Therefore, NCX may have crucial importance in the clock-like oscillator system since the NCX-mediated inward current is directly translated to membrane potential changes *via* the operation of forward mode of the exchanger. This hypothesis was repeatedly challenged and the pivotal role of Ca^2+^ clock was questioned by other authors (Noble et al., 2010; Himeno et al., 2011; DiFrancesco and Noble, 2012).

As early as 1983, Irishawa and Morad showed in elegant experiments that full inhibition of I_f_ current by caesium did not significantly influence SAN spontaneous activity arguing for mechanisms other than I_f_ (Noma et al., 1983). On the other hand, other studies suggest a fundamental role of the exchanger in normal automaticity. A low-sodium bath solution inhibited spontaneous action potentials (AP) firing in guinea-pig SAN cells *via* suppressing normal function of NCX (Sanders et al., 2006). Other studies reported that depletion of SR store by application of ryanodine markedly disturbed the normal pacemaker activity in rabbit SAN cells (Bogdanov et al., 2001). Mouse genetic models revealed that partial atrial NCX1 knock out (≈90%) caused severe bradycardia and other rhythm disorders (Herrmann et al., 2013), while complete atrial NCX knock-out completely suppressed the atrial depolarization exerting ventricular escape rhythm on the ECG (Groenke et al., 2013). The application of KB-R7943, a non-selective NCX inhibitor, also suppressed spontaneous beating in guinea-pig SAN cells (Sanders et al., 2006) however it has also marked effect on the Ca^2+^-currents. The supposed crucial role of NCX in the normal pacemaker function of SAN could not be directly investigated experimentally so far due to the lack of a selective NCX inhibitor. Recently, two novel NCX inhibitors were synthesized: ORM-10103 and ORM-10962, both showing improved selectivity without influencing I_CaL_ function (Jost et al., 2013; Kohajda et al., 2016; Oravecz et al., 2017).

In this study we confirmed the contributing role of NCX to spontaneous pacemaking by its direct pharmacological inhibition *via* the novel, selective inhibitor ORM-10962. Our data suggest that a strong crosstalk between I_f_ and NCX also exists in multicellular level, which was described and discussed by the Lakatta group earlier in single cell level (Yaniv et al., 2015). In addition, however, extending these earlier findings, we show that the effect of individual I_f_ and NCX inhibition is minimal whereas a combined inhibition acts synergistically, providing an important safety margin for secure spontaneous activity of the SAN.

## Materials and Methods

### Ethical Statement

All experiments were conducted in compliance with the *Guide for the Care and Use of Laboratory Animals* (USA NIH publication No 85-23, revised 1996) and conformed to Directive 2010/63/EU of the European Parliament. The protocols were approved by the Review Board of the Department of Animal Health and Food Control of the Ministry of Agriculture and Rural Development, Hungary (XIII./1211/2012).

### Animals

The measurements were performed in right atrial tissue obtained from young New-Zealand white rabbits from both genders weighing 2.0–2.5 kg.

### Voltage-Clamp Measurements

#### Cell Preparations

For measuring I_f_ pacemaker current, we isolated single cells from the SAN region of rabbit heart by enzymatic dissociation. The animals were sacrificed by concussion after receiving 400 IU/kg heparin intravenously. The chest was opened and the heart was quickly removed and placed into cold (4°C) solution with the following composition (mM): NaCl 135, KCl 4.7, KH_2_PO_4_ 1.2, MgSO_4_ 1.2, 4-(2-hydroxyethyl)-1-piperazineethanesulfonic acid (HEPES) 10, NaHCO_3_ 4.4, glucose 10, CaCl_2_ 1.8, (pH 7.2 with NaOH). The heart was mounted on a modified, 60 cm high Langendorff column and perfused with oxygenated and prewarmed (37°C) solution mentioned above. After washing out of blood (3–5 min) the heart was perfused with nominally Ca-free solution until the heart stopped beating (approx. 3–4 min). The digestion was performed by perfusion with the same solution supplemented with 1.8 mg/ml (260 U/ml) collagenase (type II, Worthington). After 10–12 min, the heart was removed from the cannula. The right atrium was cut and the crista terminalis and SAN region were excised and cut into small strips. Strips were placed into enzyme free solution containing 1 mM CaCl_2_ and equilibrated at 37°C for 10 min. After 10 min with gentle agitation, the cells were separated by filtering through a nylon mesh. Sedimentation was used for harvesting cells. The supernatant was removed and replaced by HEPES-buffered Tyrode’s solution. The cells were stored at room temperature in the Tyrode’s solution.

#### Measurement of Pacemaker Current (Funny Current)

For the measurement of the I_f_ current, the method of Verkerk et al. (2009) was adapted and applied. The current was recorded in HEPES-buffered Tyrode’s solution while the composition of the pipette solution was the following (in mM): KOH 110, KCl 40, K_2_ATP 5, MgCl_2_ 5, EGTA 5, HEPES 10, and GTP 0.1 (pH was adjusted to 7.2 by aspartic acid). The current was activated by hyperpolarizing voltage pulses to −120 mV from a holding potential of −30 mV. The pacemaker current was identified as ivabradine (IVA) sensitive current. The experiments were performed at 37°C.

### Fluorescent Optical Measurements

Isolated, spontaneously beating SAN cells were used for measurements. Ca^2+^ transients were measured by Fluo-4 AM fluorescent dye. Isolated cells were loaded with 5 µM dye for 20 min in room temperature in dark. Loaded cells were mounted in a low volume imaging chamber (RC47FSLP, Warner Instruments) and continuously superfused with normal Tyrode solution. Fluorescence measurements were performed on the stage of an Olympus IX 71 inverted fluorescence microscope. The dye was excited at 480 nm and the emitted fluorescence was detected at 535 nm. Optical signals were sampled at 1 kHz and recorded by a photon counting photomultiplier module (Hamamatsu, model H7828). Amplitudes of the Ca^2+^ transients were calculated as differences between systolic and diastolic values. To measure Ca^2+^ changes the cells were damaged by a patch pipette at the end of the experiment to obtain maximal fluorescence (F_max_). Ca^2+^ was calibrated using the following formula: K_d_(F−F_min_)/(F_max_−F). K_d_ of the Fluo-4 AM was 335 nM.

### Action Potential Measurements With Standard Microelectrode Technique

We have chosen multicellular preparations for action potential measurements for three reasons: 1) all of the ion channels remained intact (current density, kinetics) because of the lack of enzymatic dissociation, thus providing more precise estimates of the ratio between the currents, 2) since the SAN cells are surrounded with atrial cells having a more negative resting membrane potential, the electrotonic coupling may intimately influence the SAN cells. It may have great importance since the I_f_ current markedly increases as the membrane potential drops to more negative values (Morad and Zhang, 2017), (3) the action potential frequency was very stable with a cycle-length variability lower than 5 ms.

SAN regions obtained from right atria were mounted in a tissue chamber superfused with oxygenated Locke’s solution at 37°C. A conventional microelectrode technique was performed as previously described (Kormos et al., 2014; Kohajda et al., 2016; Oravecz et al., 2017). In the case of SAN, the action potentials were empirically found in the right atrium. SAN action potentials were verified by the maximum upstroke speed, which had to be lower than 15 V/s, the resting membrane potential (>−60 mV), and a clear diastolic depolarization (DD). Efforts were made to maintain the same impalement throughout each experiment. If impalement became dislodged, however, electrode adjustment was attempted and, if the action potential characteristics of the re-established impalement deviated by less than 5% from those of the previous measurement, the experiment was continued. When this 5% limit was exceeded, the experiment was terminated and all data were excluded from the analyses.

Action potential durations were measured at 90, 50, and 25% of repolarization. The maximal diastolic potential was defined as the most negative potential reached during the repolarization. The take off potential is the most negative point of the AP upstroke. DD was defined as the interval between the maximal diastolic potential and take off potential. The DD slope was calculated as ΔV_m_/Δt between these points. The cycle length was calculated as the peak-to-peak interval between two consecutive APs. The phase 0 depolarization velocity was defined as the maximum of the first derivative of the AP during the upstroke.

### Modeling

To complement the experiments, we performed mechanistic computational modeling using the Yaniv *et al*. model of rabbit SAN cells (Yaniv et al., 2013b). The differential equations of the model were solved using a forward Euler scheme. Simulation results were analyzed when the system had converged to a cyclic steady-state.

### Statistical Analysis

All data are expressed as mean ± standard error (SEM). Statistical analysis was performed with Student’s *t-*test and ANOVA with Bonferroni *post-hoc* test. The results were considered statistically significant when p was < 0.05. In the case of action potential experiments, all recordings were obtained from different hearts.

### Materials

With the exception of ORM-10962 (ORM) (from Orion Pharma, Espoo, Finland), and Fluo-4 AM (Thermo Fisher Scientific, Waltham, MA, USA), all chemicals were purchased from Sigma-Aldrich Fine Chemicals (St. Louis, MO, USA). ORM was dissolved in dimethyl-sulfoxide (DMSO) to obtain a 1 mM stock solution. This stock solution was diluted to reach the desired final concentration (DMSO concentration not exceeding 0.1%) in the bath.

## Results

### ORM-10962 Has No Effect on Funny Current

**Figure 1:**
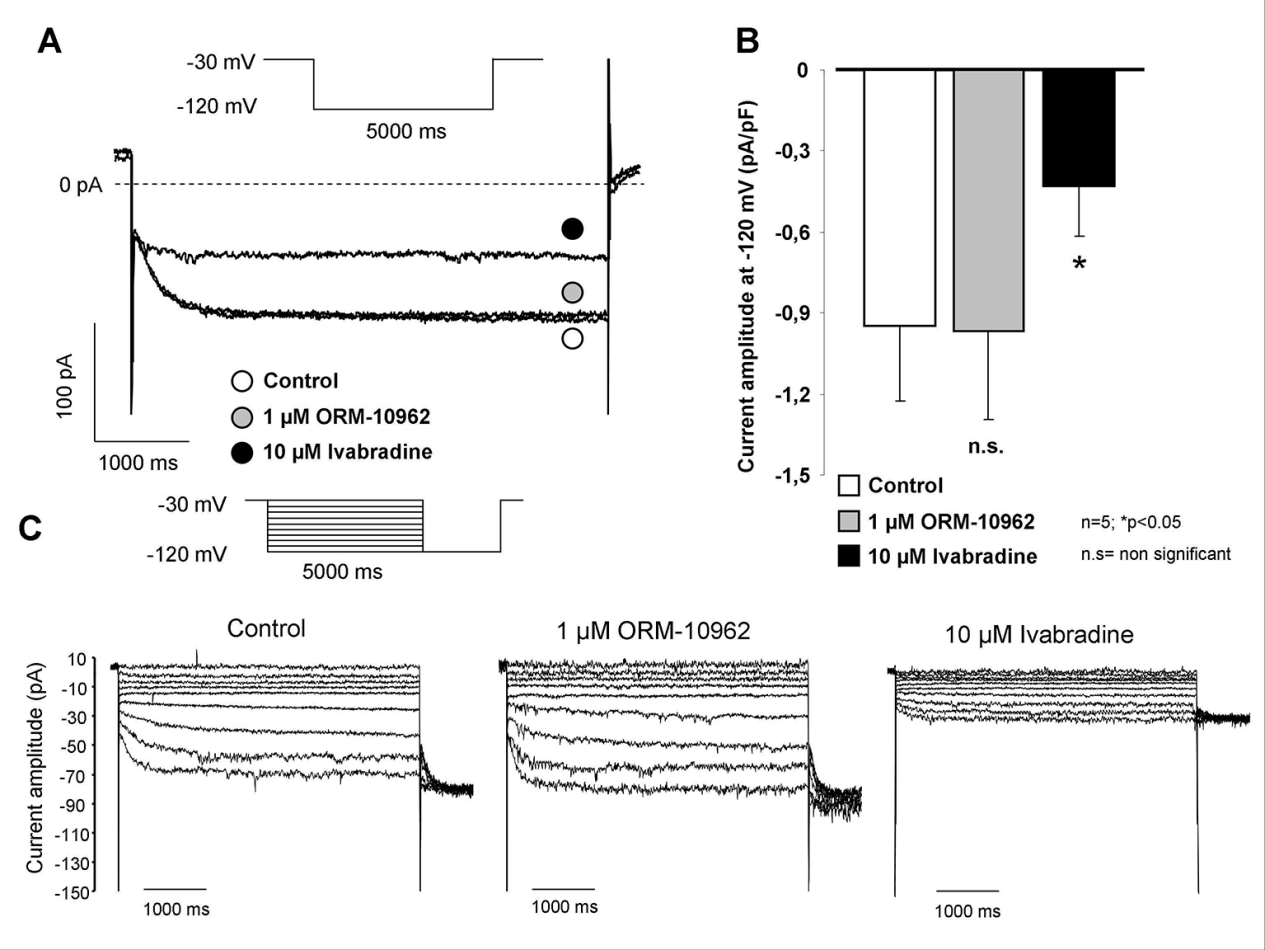
Investigation of the possible effect of ORM-10962 (ORM) on funny current (I_f_) in isolated sinus node (SAN) cells. The hyperpolarization activated I_f_ was elicited by 5,000 ms long rectangle pulse potentials to −120 mV from a holding potential of −30 mV. As representative current traces indicate, the current amplitude after ORM application (gray circle) was identical with the control (open circle). The considerable effect of 10 µM ivabradine (IVA) verified that the elicited current was indeed I_f_ **(A**, **B)**. Original traces in panel **(C)** represent the absence of ORM effects on current-voltage relationship of I_f_ by applying hyperpolarization pulses from −120 mV to −30 mV with 10 mV increments.

**Figure 1** shows the measurement of I_f_ in isolated SAN cells by applying the whole cell configuration of the patch clamp method. The selectivity of ORM on Na^+^-, Ca^2+^-, and major K^+^ currents was tested in a previous study from our laboratory (Kohajda et al., 2016). However, its potential effect on the I_f_ current was not investigated in that previous study. As **Figures 1A**, **C** show a slowly developed current at negative hyperpolarizing membrane potential (from −30 to −120 mV), which was not altered by application of 1 µM ORM (**Figure 1B**). In contrast, it was markedly inhibited by 10 µM IVA (69.3 ± 3.4%), a well known inhibitor of I_f_.

### Na^+^/Ca^2+^ Exchanger Inhibition Exerted Moderate Bradycardic Effect on Sinus Node Tissue

**Figure 2:**
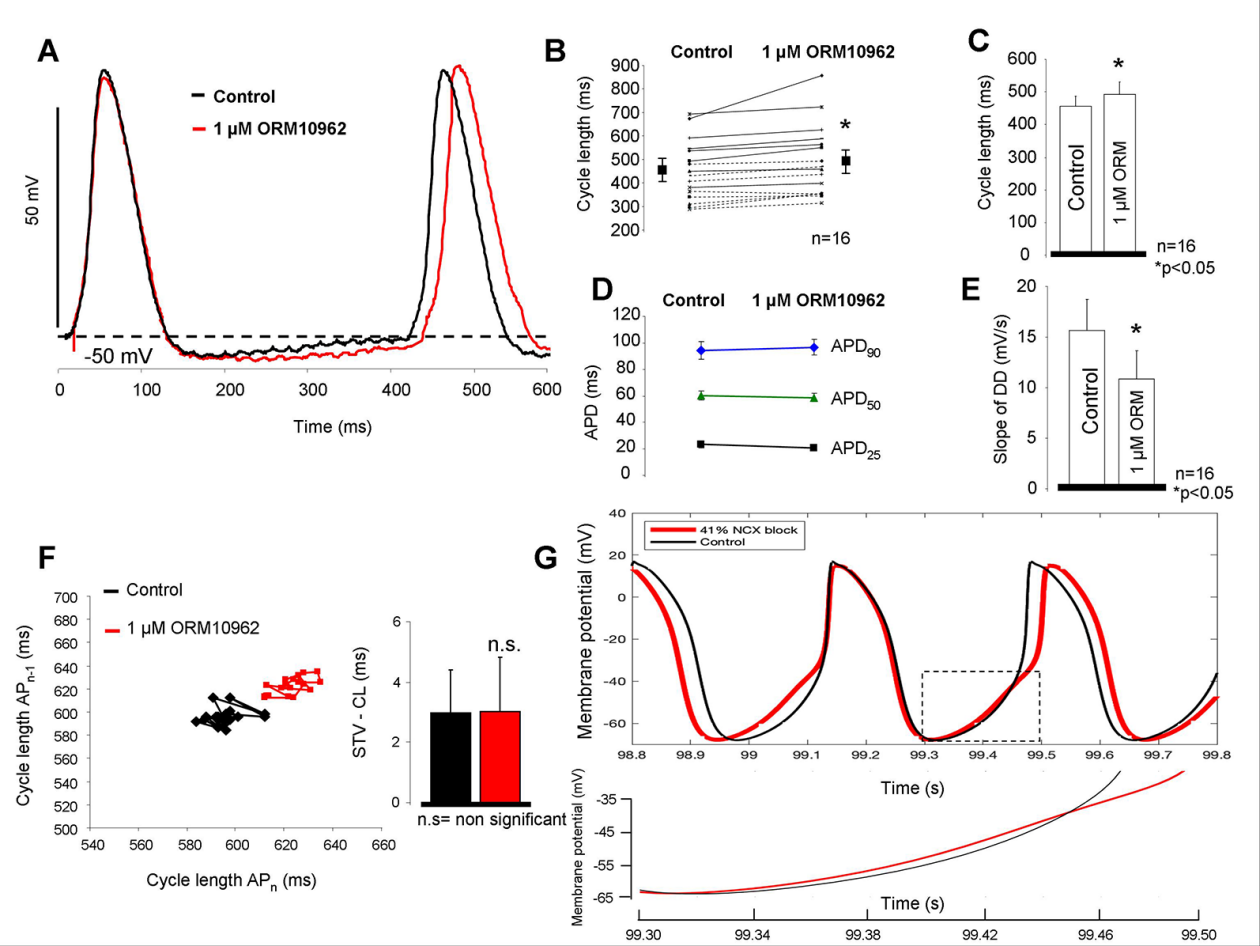
Estimation of the effect of selective Na+/Ca2+ exchanger (NCX) inhibition on sinus node (SAN) tissue. As representative action potential traces **(A)** as well as individual experiments **(B)** and bar graphs **(C)** indicate, application of 1 µM ORM-10962 (ORM) exerted a slight but statistically significant bradycardic effect on SAN tissue. The action potential duration (APD) did not change during the experiment **(D)**, however the slope of the spontaneous depolarization was considerably decreased **(E)**. 30 consecutive cycles were analyzed to estimate the pacing rate variability. Poincaré-plot and bar graphs depict that ORM did not alter the short-term cycle length (CL) variability **(F)**. The Yaniv SAN cell model predicts 41% NCX inhibition to meet with the observed bradycardic effect of 1 µM ORM. The inset illustrates the reduced slope during late diastolic depolarization (DD) when 41% NCX inhibition was applied (red curve) **(G)**.

**Figure 2** summarizes the effect of selective NCX inhibition by ORM on the spontaneous automaticity in SAN. Following application of 1 µM ORM a moderate but significant lengthening effect on the CL was observed (455.6 ± 32 ms *vs.* 493.0 ± 38 ms; Δ = 8.1 ± 1.8% p < 0.05, n = 16/16 hearts; **Figures 2A**–**C**) without any influence on the action potential duration (APD) (94.3 ± 6.7 ms *vs.* 96.7 ± 5.9 ms; **Figure 2D**). The slope of the diastolic depolarization phase was significantly reduced after ORM application (15.7 ± 3.1 mV/s *vs.* 10.9 ± 2.8 mV/s; n = 14/14; p < 0.05 **Figure 2E**) while the CL variability remained unchanged (7.6 ± 1.2 ms *vs.* 8.1 ± 1.3 ms; **Figure 2F**). The slope of phase 0 AP depolarization was identical during control and ORM experiments (11.2 ± 2.7 V/s *vs.* 12.5 ± 2.3 V/s). The preparations maintained the stable frequency in the time control experiments when DMSO was applied (440 ± 36.1 ms *vs.* 445 ± 37.6; n = 4). In the computational SAN action potential model (Yaniv et al., 2013b), we identified the degree of NCX current suppression required to obtain a similar CL increase as was experimentally measured. Forty-one percent of NCX inhibition was required to obtain 8% CL increase which was equal with the CL change observed experimentally (**Figure 2G**).

### Na^+^/Ca^2+^ Exchanger Inhibition Slightly Increased the Diastolic Ca^2+^ Level in Isolated Sinus Node Cells

**Figure 3:**
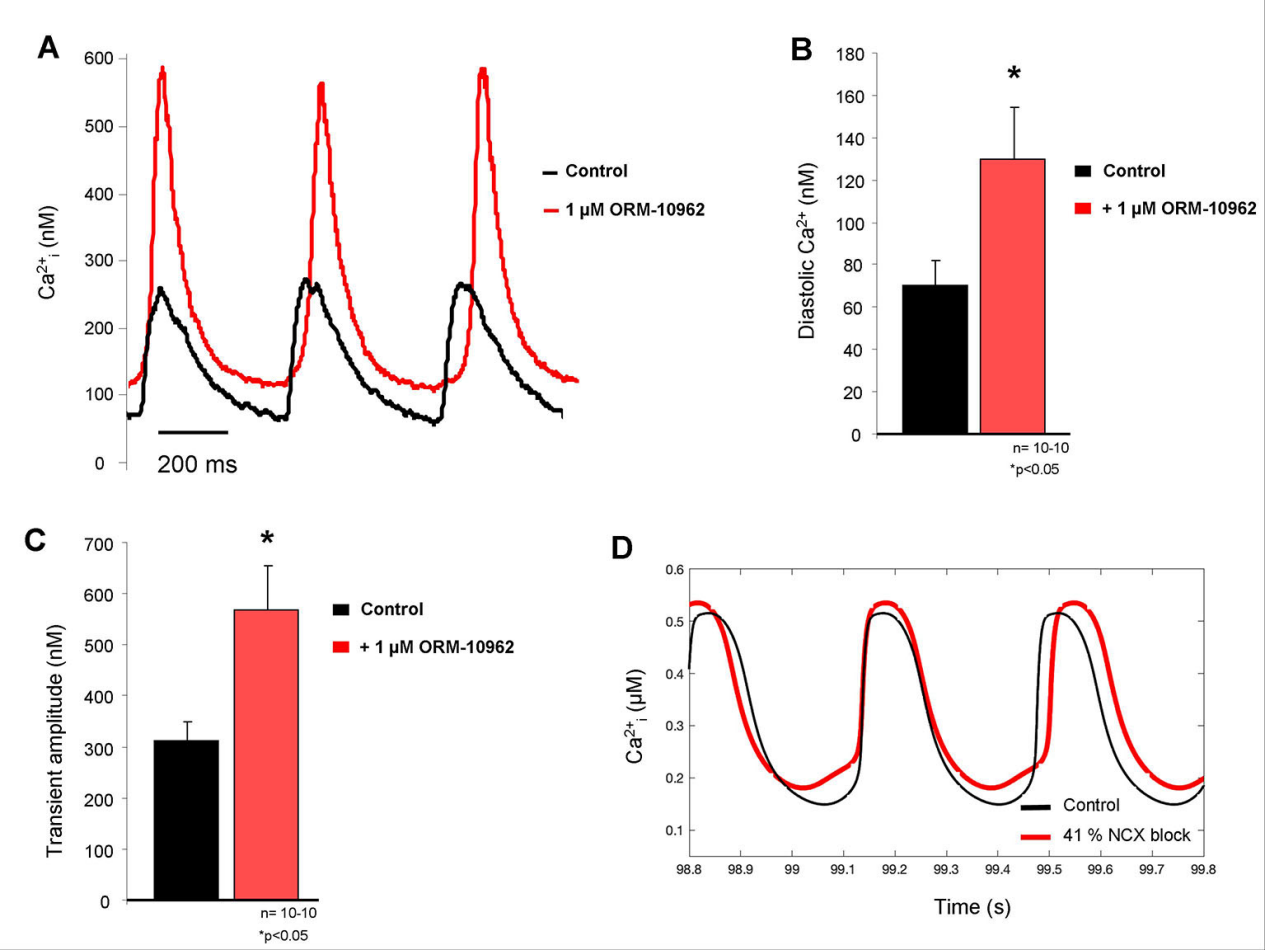
**(A)** Representative Ca^2+^ transient traces from spontaneously contracting isolated sinus node (SAN) cells under control conditions (black trace) and after 1 μM ORM-10962 (ORM) application (red trace). As original fluorescent data as well as bar graphs indicate, a slight but significant increase of the diastolic Ca^2+^ level **(B)** with increased transient amplitude **(C)** was observed. In line with experimental data, the Yaniv SAN cell model predicted similar diastolic Ca^2+^ gain after 41% Na+/Ca2+ exchanger (NCX) inhibition **(D)**, however the increase of transient amplitude is much more pronounced during the experimental results.

The diastolic Ca^2+^ level increased in isolated SAN cells after ORM treatment (70 ± 11 nM *vs.* 130 ± 24 nM; p < 0.05, n = 10; **Figures 3A**, **B**), the effect was similar than was predicted by the Yaniv et al. SAN model (**Figure 3D**). In contrast to the model prediction, we found considerable increase in the transient amplitude (312 ± 37 nM *vs.* 568 ± 85 nM; p < 0.05, n = 10 **Figure 3C**), which was nearly doubled (82.1 ± 22%) in response to ORM application compared to the control value.

### The Concomitant Application of Ivabradine and ORM-10962 Revealed Coupled Frequency Control Between Funny Current and Na^+^/Ca^2+^ Exchanger Measured in Sinus Node Tissue

**Figure 4:**
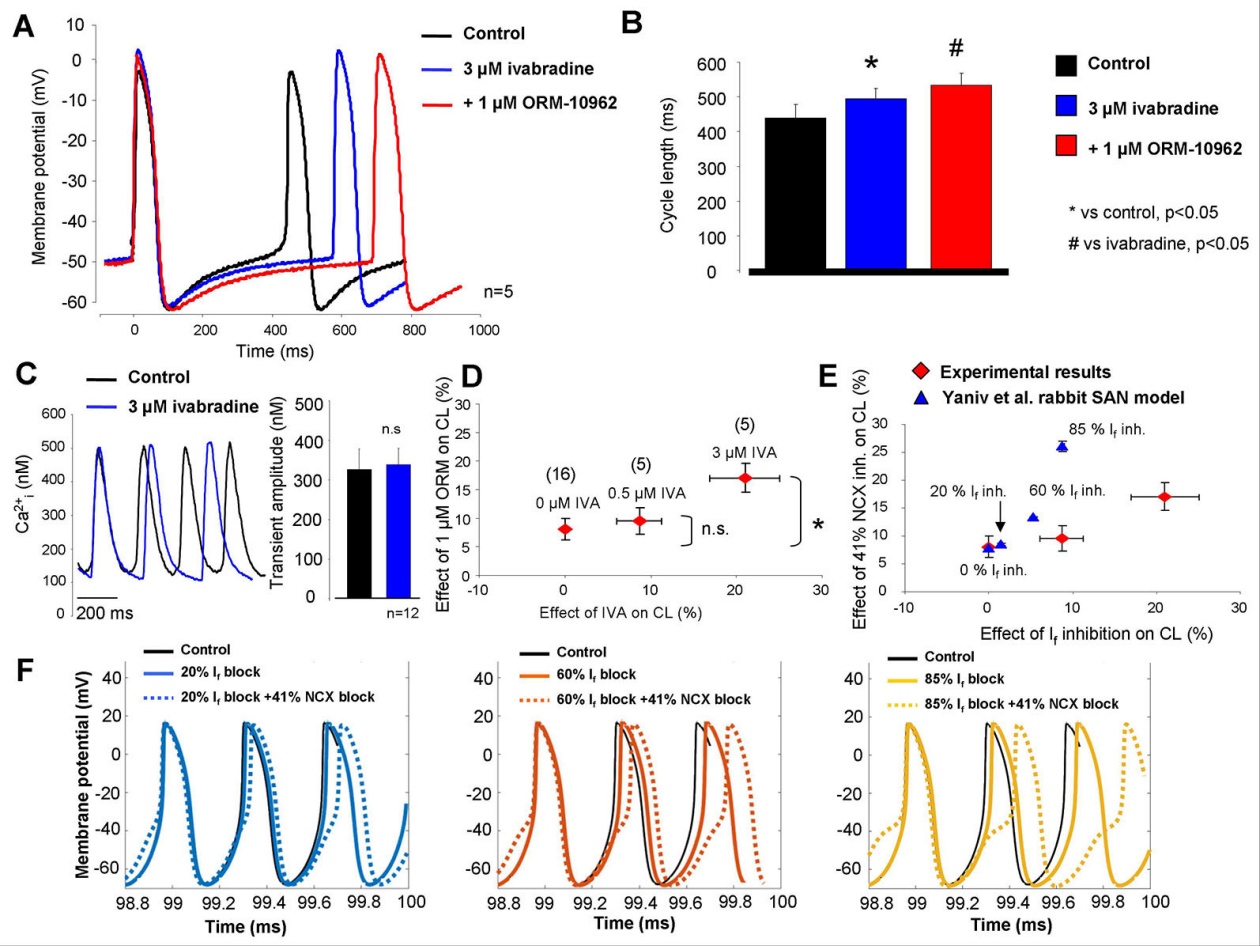
Combined inhibition of Na+/Ca2+ exchanger (NCX) and I_f_ in sinus node (SAN). As original SAN action potentials and bar graphs report **(A, B)**, 1 μM ORM-10962 (ORM) (red trace) exerted an increased effect after 0.3 μM ivabradine (IVA) pretreatment (blue trace). Panel **(C)** represent Ca^2+^ transients measured from isolated SAN cells under control condition (black trace) and in the presence of 3 μM IVA (blue trace). We found identical Ca^2+^ levels as a result of IVA treatment. In panel **(D)**, the dose dependent effect of IVA (abscissa) on SAN cycle length (CL) was plotted against the effect of consecutive application of 1 μM ORM on CL (ordinate). As was previously described in **Figures 2A**–**C**, 1 μM ORM has ≈8% effect without IVA. In the presence of 0.5 and 3 μM IVA, the ORM-induced reduction of pacing rate was gradually increased. The numbers in parentheses indicate the corresponding n. The experimental results (red) are compared with the Yaniv SAN cell model (blue) in panel **(E)**. Based on a previous study, 0.5 and 3 µM IVA were represented by 20 and 60% funny current (I_f_) inhibition in the presence of constant 41% NCX inhibition. Panel **(F)** represents the modeling results of combined I_f_-NCX block. In the three panels, I_f_ was inhibited by varying degrees (straight lines) and combined with 41% NCX inhibition (dotted lines) yielding an increasing NCX inhibition effect on CL as I_f_ inhibition increases.

In the next set of experiments, IVA and ORM were subsequently applied to study a possible coupling between I_f_ and NCX. The effect of 1 µM ORM was substantially larger when I_f_ was previously inhibited (**Figures 4A**, **B**). Ca^2+^ transient measurements from spontaneously contracting SAN cells showed identical amplitudes (327 ± 23 nM *vs.* 337 ± 42 nM; n = 12) as well as diastolic Ca^2+^ levels (89 ± 22 nM *vs.* 85 ± 13 nM; n = 12) between control and 3 µM IVA (**Figure 4C**). A clear, gradual increase of ORM effect on the CL was observed with combined increasing concentration of IVA (1 µM ORM effect in the presence of 0 µM IVA: 8.1 ± 1.88%; in the presence of 0.5 µM IVA: 9.6 ± 2.3%; in the presence of 3 µM IVA: 17.1 ± 2.5%; **Figure 4D**). The ORM effect in the presence of 0.5 µM IVA did not differ significantly from the control, where 0 µM IVA was applied (8.1 ± 1.88% *versus* 9.6 ± 2.3%). In contrast, ORM effect was significantly larger on the CL in the presence of 3 µM IVA, compared with the control where IVA was not applied (8.1 ± 1.88% *versus* 17.1 ± 2.5%; p < 0.05, ANOVA, Bonferroni *post hoc* test). IVA significantly increased the CL both in 0.5 and in 3 µM concentrations (p < 0.05, ANOVA, Bonferroni *post hoc* test). In **Figure 4E**, we compare modeling and experimental results. In the Yaniv et al. model, based on a previous study (Bois et al., 1996), I_f_ inhibition was varied between 0%/20%/60% block (corresponding to 0, 0.5, and 3 µM IVA). Larger, 85% inhibition was only set in the model, since experimental application of 10 µM IVA is not feasible because of the marked I_Kr_ inhibition which can also reduce automaticity. The NCX inhibition was 41% in all cases. As **Figure 4E** shows, the modeling results do not match the experiments quantitatively, however they show a similar tendency (when I_f_ block increases, the same NCX inhibition causes larger CL prolongation) with markedly steeper correlation.

**Figure 4F** shows original modeling traces in the presence of 20% (left panel), 60% (middle panel), and 85% (right panel) I_f_ inhibition when NCX inhibition was 41% in all cases. The action potential modeling demonstrates an increased CL prolongation effect of NCX inhibition as I_f_ suppression becomes stronger, however, in contrast to the model prediction the steepness of NCX inhibition-induced CL increase was considerable flatter during experiments.

### I_Kr_ Inhibition-Induced Bradycardia Did Not Facilitate the Effect of Selective Na^+^/Ca^2+^ Exchanger Inhibition on Cycle Length in Sinus Node Tissue

**Figure 5:**
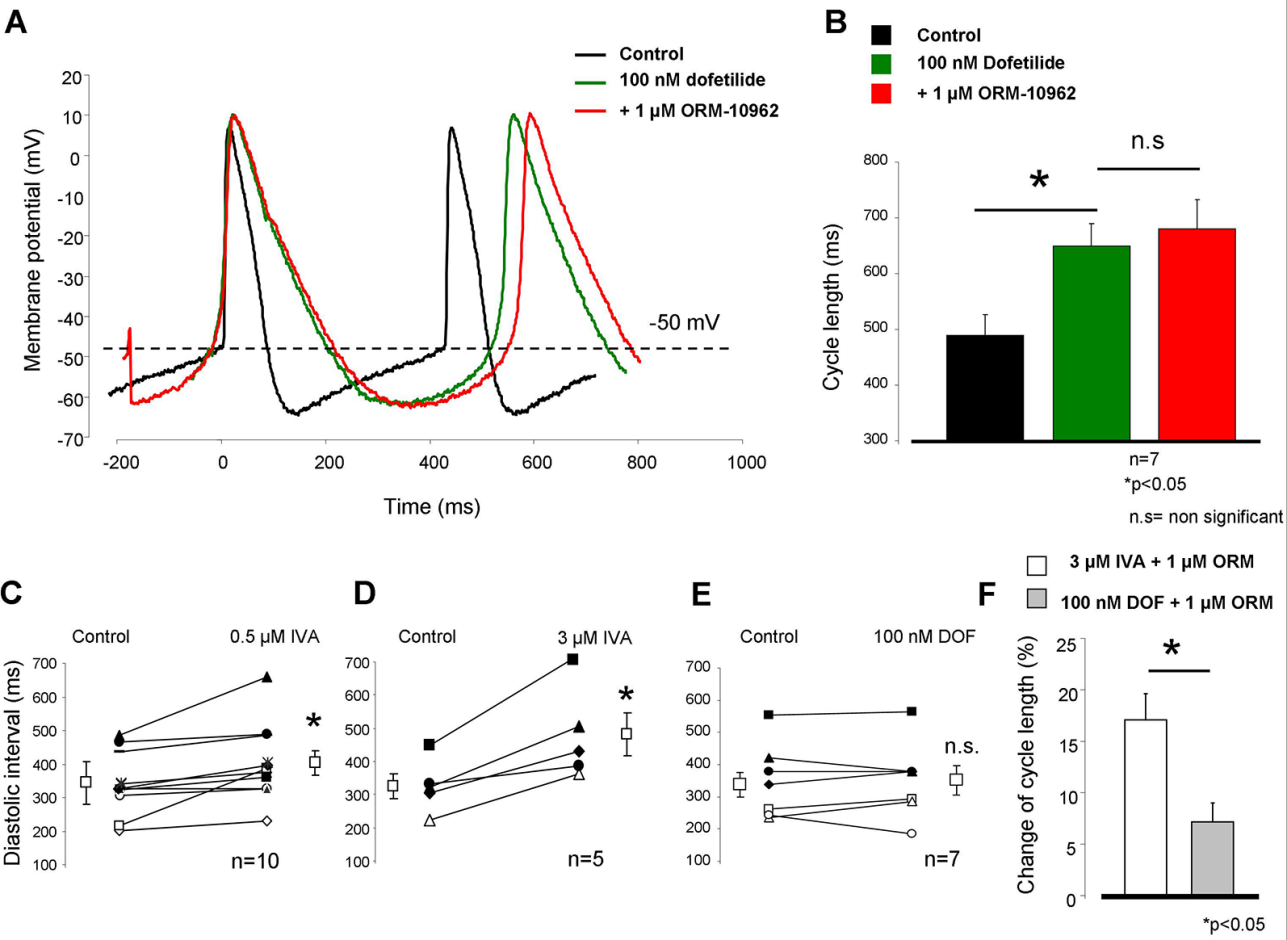
Sinus node (SAN) frequency was decreased by complete I_Kr_ inhibition by 100 nM dofetilide administration. The control cycle length (CL) (black trace) largely increased after dofetilide (green trace). However, the effect of 1 µM ORM-10962 (ORM) did not change compared with the individual effect (red trace, panels **A, B, F)**. The main difference between ivabradine (IVA) and dofetilide (DOF)-induced bradycardia is that 0.5 and 3 µM IVA markedly increased the diastolic interval **(C**, **D)**, which remained unchanged in the case of I_Kr_ inhibition by DOF **(E)**.

We investigated how bradycardia induced by a mechanism which does not directly involve the inward depolarizing currents (I_f_ and NCX) would influence the effect of NCX inhibition. Full I_Kr_ block induced by 100 nM dofetilide (DOF) markedly increased the CL of SAN AP (control: 489.3 ± 31 ms → 100 nM dofetilide: 649.1 ± 40.2 ms). This degree of increase of CL was due to the lengthening of APD without changing the DI. The subsequent application of 1 µM ORM exerted a similar effect (1 µM ORM-10962: 679.6 ± 52.6 ms; n = 7/7 hearts; **Figures 5A**, **B**), compared with results obtained after individual administration presented in **Figure 2** (7.2 ± 1.8% *vs.* 8.1 ± 1.8%, **Figure 5F**). It is important that the effect of DOF on CL was nearly similar to 3 µM IVA (32.9 ± 6.7% *vs.* 20.9 ± 4.1%). However, the major difference was that the DOF-mediated increase in CL was practically entirely an APD increase-induced effect (APD_90_: 94.4 ± 3 ms *vs.* 187 ± 7.1 ms; p < 0.05, n = 7; diastolic interval (DI): 338.3 ± 39 ms *vs.* 352.7 ± 44.6 ms, n = 7) while the IVA influenced only the DI without affecting the APD_90_ (**Figures 5C**–**E**). In contrast, both NCX inhibition by ORM and I_f_ inhibition by IVA increased the CL due to lengthening of the time of the DI by decreasing its slope. When ORM was applied in combination with DOF the increase of the CL was not additive.

### Suppression of Ca^2+^ Increases the Effect of I_f_ Inhibition on Cycle Length in Sinus Node Tissue

**Figure 6:**
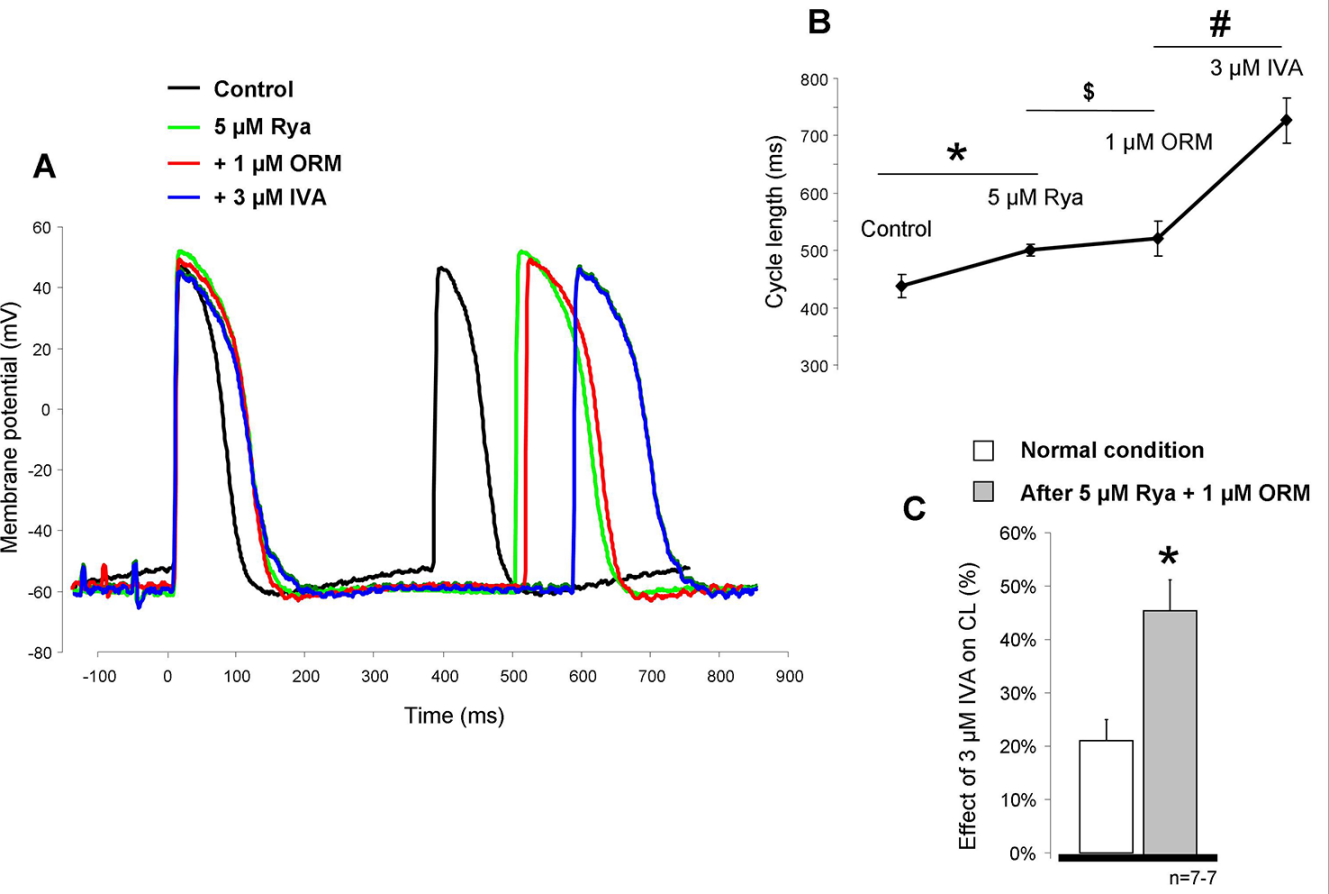
The potential influence of Ca^2+^ handling suppression by 5 µM ryanodine (RYA) (green line) and 1 µM ORM-10962 (red line) on the effect of funny current (I_f_) reduction was investigated. As representative curves panel **(A)** and diagram panel **(B)** report, both drugs increased the cycle length (CL) of the action potential. Statistical analysis was achieved by ANOVA. * means RYA compared to control, $ means ORM-10962 (ORM) compared to RYA, and # means ivabradine (IVA) compared to ORM. Comparison of the effect of 3 µM IVA on the CL under normal condition (i.e., without any other inhibitors) and after 5 µM RYA + 1µM ORM shows that the same dose of IVA has markedly increased effect when the contribution of Na+/Ca2+ exchanger (NCX) is reduced by concomitant application of RYA and ORM panel **(C)**.

In the next set of experiments, we investigated the potential effect of suppression of SR Ca^2+^ release on the effect of IVA (**Figure 6A**). The aim was to minimize the depolarizing activity of the Ca^2+^ release-induced augmentation of the forward NCX by application of 5 µM ryanodine (RYA) after the control recording. This caused a significant CL prolongation (437.8 ± 20.3 ms *vs.* 499.8 ± 10.4 ms; p < 0.05, n = 6/6). The subsequently applied 1 µM ORM-10962 marginally but statistically significantly increased the CL (499.8 ± 10.4 ms *vs.* 520.8 ± 29.9 ms; p < 0.05; n = 6/6). However, further 3 µM IVA markedly and significantly augmented the CL of the SAN preparations (520.8 ± 29.9 ms *vs.* 726.6 ± 39.8 ms; p < 0.05, n = 6/6; **Figure 6B**). In the **Figure 6C** we compared the IVA effects under normal condition (i.e., in the absence of any other inhibitors—20.9 ± 4.1%) and in the presence of RYA+ORM. As bar graphs in **Figure 6C** show, the IVA exerted markedly larger CL prolongation in the presence of RYA+ORM (42.4 ± 5.7%, p < 0.05, Student’s T-test).

### Decrease of [Ca^2+^]_O_ Increases the Effect of Funny Current Inhibition on Cycle Length in Sinus Node Tissue

**Figure 7:**
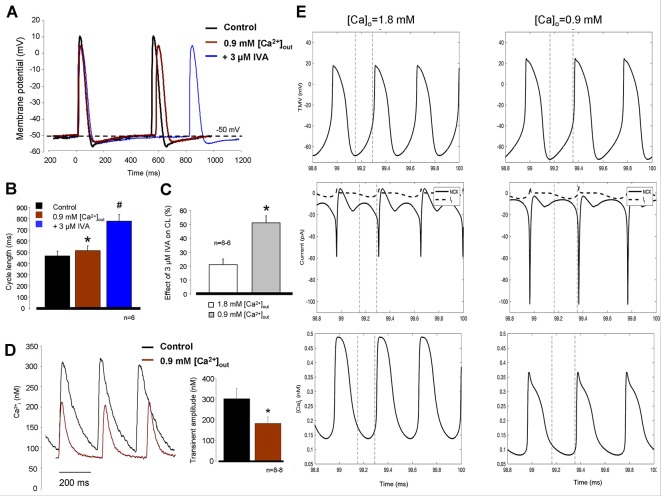
Decreased extracellular Ca^2+^ solution (0.9 mM) was used to suppress the intracellular Ca^2+^ cycling and therefore Na+/Ca2+ exchanger (NCX). The effect of hypocalcemic solution on the cycle length (CL) was marginal (**A, B**, brown trace) however the subsequently applied 3 µM ivabradine (IVA) (blue trace) caused considerable prolongation in the CL. Comparison of the IVA effect in the presence of normal (1.8 mM—white column) *versus* low (0.9 mM—gray column) CaCl_2_ on panel **(C)** demonstrates nearly doubled effect of IVA on the CL in response of Ca^2+^ reduction **(C)**. * means 0.9 mM [Ca^2+^]_o_ compared to control, # means IVA *versus* 0.9 mM [Ca^2+^]_o_. Original traces measured from isolated sinus node (SAN) cells in panel **(D)** demonstrate that 0.9 mM [Ca^2+^]_o_ (brown trace) significantly decreased the transient amplitude without significant action on diastolic Ca^2+^ levels. **(E)** Modeling simulation of action potentials (upper traces), NCX currents (middle traces, solid lines), I_f_ currents (middle traces, dashed lines), and global Ca^2+^ transients (lower traces) in the present of normal (1.8 mM), external Ca^2+^ (left column), and 0.9 mM [Ca^2+^]_o_ (right column). The results indicate decreased transient amplitude coupled with smaller NCX current in the late diastolic depolarization (DD) with maintained I_f_ current magnitude in the presence of 0.9 mM [Ca^2+^]_o_.

We further tested the coupling between Ca^2+^ handling and I_f_ on CL control. Reduced [Ca^2+^]_o_ (0.9 mM) external solution was selected to achieve this goal since in this concentration, the CL was only slightly reduced (10.3 ± 3.7%). We found that the reduced extracellular Ca^2+^ slightly increased the CL which was further increased after application of 3 µM IVA (control: 469 ± 39.5 ms → 0.9 mM [Ca^2+^]_o_: 515.8 ± 40.8 ms → 3 µM IVA: 777 ± 58.7 ms; p < 0.05, n = 6/6 hearts, **Figures 7A, B**). We compared again the effects of IVA on the CL under normal condition (i.e., 1.8 [Ca^2+^]_o_) and in the presence of low external Ca^2+^ (0.9 mM [Ca^2+^]_o_). As bar graphs in **Figure 7C** demonstrates the IVA has markedly improved effect when extracellular Ca^2+^ is low compared with normal Ca^2+^ settings (51.1 ± 5.1% *versus* 20.99 ± 4.1%, p < 0.05; Student’s t-test). **Figure 7D** represents Ca^2+^ transient measurements from spontaneously contracting isolated cells. We can observe that the application of 0.9 mM [Ca^2+^]_o_ significantly decreased the transient amplitude (295 ± 52 nM *vs.* 185 ± 32 nM; p < 0.05, n = 8) which may reflect decreased Ca^2+^ influx, SR Ca^2+^ release which may decrease the NCX current and thus attenuate the compensating capacity of NCX. The diastolic Ca^2+^ also significantly decreased (127 ± 33 *vs.* 64 ± 10 nM; p < 0.05, n = 8). We addressed this question by using mechanistic modeling (Yaniv et al., 2013b). Left column of **Figure 7E** represents action potentials (upper traces), NCX and I_f_ current kinetics (middle traces), and global Ca^2+^ transients (lower traces) under normal condition. Upon application of 0.9 mM [Ca^2+^]_o_ (right column) the CL slightly reduced, the integral of NCX current under the late phase of DD decreased while the magnitude of I_f_ current did not changed. The amplitude of the global transient decreased in similar extent as was obtained from SAN cell experiments.

### Concomitant Inhibition of Na^+^/Ca^2+^ Exchanger and Funny Current Increases the Cycle Length Variability in Sinus Node Tissue

The short term CL variability (CLV) was calculated by the analysis of CLs of N = 30 consecutive action potentials by applying the following formula:

$$\text{STV}=\text{Σ}\left(\text{CL};\text{i}+1-\text{CL};\text{i}\right)/\left({\text{n}}_{\text{beats}}x\sqrt{2}\right).$$

**Figure 8:**
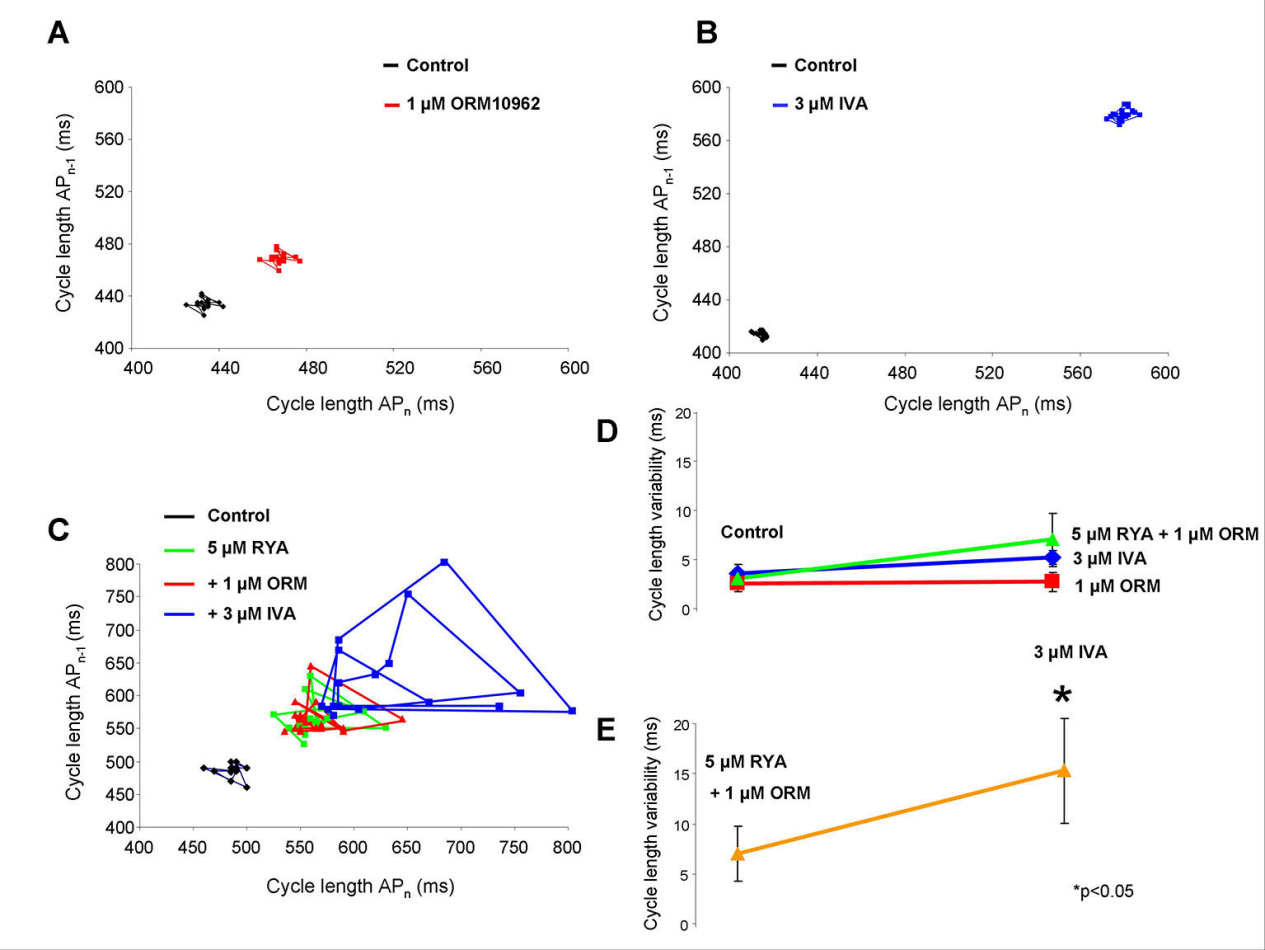
The effect of suppression of diastolic depolarization (DD) currents [Na+/Ca2+ exchanger (NCX) and I_f_] on short time cycle length (CL) variability. When 1 µM ORM-10962 panel **(A)** or 3 µM ivabradine (IVA) panel **(B)** were applied, the variability did not change significantly. Instead, the CL was prolonged. Comparison of the effect of 1 µM ORM-10962 (ORM) (red line), 5 µM ryanodine (RYA) (blue line), and 5 µM RYA + 1 µM ORM (green line) on the CL variability shows that these compound do not change the variability significantly panel **(D)**. In contrast IVA administration after the application of RYA+ORM caused considerably increased variability panel **(E)** as the Poincaré-plot (panel **C**, blue line) shows.

One micrometer of ORM-10962 and 3 µM IVA individually prolong the CL without considerable influence on the CL variability (see the area covered in **Figures 8A, B**). The subsequent application of 5 µM RYA (**Figure 8C**, green line) and 5 µM RYA + 1 µM ORM-10962 (**Figure 8C**, red line) showed a tendency to increase the CL variability, however it proved not to be statistically significant. In contrast, additionally adding 3 µM IVA markedly and statistically significantly enhanced the variability parallel with the CL increase, when the Ca^2+^ release and NCX activity were suppressed (**Figure 8C**, blue line). As **Figures 8D**, **E** show, the CL variability exerts similar results as the CL measurements: individual inhibition of NCX (2.53 ± 0.8 ms *vs.* 2.71 ± 0.9 ms; n = 16/16; red line) and I_f_ (3.6 ± 0.9 ms *vs.* 5.19 ± 0.7 ms; n = 5/5; blue line) or Ca^2+^ handling suppression (3.03 ± 0.87 ms *vs.* 7.0 ± 2.73 ms, n = 7/7; green line) do not alter significantly the CLV while the variability was largely increased if IVA was administrated in the presence of reduced Ca^2+^ cycling activity (7.0 ± 2.73 ms *vs.* 15.29 ± 5.6 ms; orange line).

## Discussion

The aim of this study was to verify and estimate the possible contribution of NCX function in SAN automaticity by direct selective pharmacological inhibition. Furthermore, we evaluated the functional consequences of the previously mentioned (Yaniv et al., 2015) I_f_-NCX coupling in multicellular tissue level. In this study, we provided evidence for the first time regarding the essential role of NCX in spontaneous automaticity of the SAN by selective pharmacological inhibition. In addition, we described its functional interaction with I_f_. This interaction between the DD currents is based on the following experimental results: i) 3 µM IVA has moderate effects on CL (~21%) and CLV (Δ ~ 2 ms), ii) 1 µM ORM has marginal effects on CL (~8%) and no change on CLV, iii) Ca^2+^ cycling suppression by 1 µM ORM + 5 µM RYA has moderate effects on CL (~19%) and CLV (Δ ~ 4 ms), iv) increasing I_f_ inhibition augments the effect of a fixed ORM dose (1 µM) on CL (~ 8 to 17%), v) the effect of 3 uM IVA is enhanced when Ca^2+^ cycling was previously suppressed (from ~ 20 to 42%).

### ORM-10962 Does Not Inhibit the Funny Current

The effectiveness and selectivity of ORM-10962, a novel, potent NCX inhibitor was investigated in detail in our previous studies (Kohajda et al., 2016; Oravecz et al., 2017). In these studies, it was shown that ORM inhibited both forward and reverse mode NCX with an IC_50_ values of 55 and 67 nM without changing the I_Ca_, I_Na_, I_K1_, I_Kr_, I_Ks_, I_to_, and I_Na_/_K_ pump currents even at high (1 μM) concentrations. However, the I_f_ current was not investigated. The present study demonstrates that 1 µM ORM did not influence I_f_ (**Figure 1**) in the presence of high Ca^2+^ buffering, which means that ORM is a suitable tool for the evaluation of NCX in SAN automaticity, however the indirect effects related with ORM-induced Ca^2+^ elevation (without Ca^2+^ buffering) may influence the I_f_ indirectly (Mattick et al., 2007).

### Na^+^/Ca^2+^ Exchanger Inhibition Slightly Decreases Sinus Node Firing Rate

We found slight, but statistically significant reduction in the spontaneous firing rate in SAN tissue which is the consequence of the reduced rate of the DD (**Figure 2**). This result is a direct evidence and verification for the contribution of the inward NCX in rhythm generation. Previous studies (Yaniv et al., 2013a; Yaniv et al., 2015) reported that not only the increase of CL, but the parallel increase of pacing variability reports the uncoupling of the I_f_-NCX and the destabilization of the DD. Since in our experiments the CL slightly increased while the pacing rate variability did not change, we conclude that the individual NCX inhibition did not cause considerable uncoupling of I_f_-NCX.

The rate of forward NCX inhibition by using 1 µM ORM was estimated to ≈90% by applying conventional ramp protocol in the presence of ≈160 nM [Ca^2+^]_i_ in canine ventricular myocytes in our previous study (Kohajda et al., 2016). The actual ratio of inhibited NCX which corresponds with the observed CL changes was calculated to 41% by using the Yaniv SAN cell model (Yaniv et al., 2013b). It is important to note, that in our previous study (Oravecz et al., 2017) we have demonstrated that the extent of NCX inhibition (*via* ORM-10962) is decreased when the intracellular Ca^2+^ is intact (i.e., in the presence of Ca^2+^ transient). The underlying mechanism could be asymmetrical block between two modes, autoregulation of the Ca^2+^_i_ or by preserved inducibility of forward NCX by elevated Ca^2+^_i_.

### Na^+^/Ca^2+^ Exchanger Inhibition Markedly Increases the Ca^2+^_I_ Level

The selective NCX inhibition caused similar diastolic Ca^2+^ changes compared to the Yaniv model predicted, however, in contrast with modeling, we found markedly increased Ca^2+^ transient amplitude which is generally expected after decreased rate of Ca^2+^ extrusion (**Figure 3**). The observed quantitative discrepancy between experiments and modeling may indicate that the extent of NCX inhibition in the experiments could be larger than 41%.

We can speculate that the increasing intracellular Ca^2+^ is known to facilitate the inactivation of the L-type Ca^2+^ current as a part of the autoregulation (Eisner et al., 1998; Eisner et al., 2000). The gain of the [Ca^2+^]_i_ may indirectly shortens the CL which means two parallel, counteracting effect of selective NCX inhibition: the inhibition of the inward NCX current may reduce the actual frequency by suppressing its contribution in the DD, however it is partially compensated for the CL abbreviating effect of increased [Ca^2+^]_i_. Furthermore, the I_f_ may also contribute in the limitation of the ORM effect: i) a theoretical possibility exists that ORM-induced Ca^2+^ elevation may increase the I_f_, however this was ruled out by a previous work (Zaza et al., 1991). ii) It was reported that SAN myocytes express Ca^2+^-activated adenylate cyclase isoform, which might raise cAMP (and I_f_) in response to NCX blockade (Mattick et al., 2007).

### The Moderate Bradycardic Effect of Na^+^/Ca^2+^ Exchanger Inhibition May Be Explained by Funny Current-Na^+^/Ca^2+^ Exchanger Coupling

However, one may speculate after considering the crucial role of NCX in the coupled-clock theory, why the NCX inhibition-induced “bradycardia” exerted a relatively low influence. Using genetic mouse models Gao *et al*. claimed that partial ablation of NCX (≈70–80%) using an αMHC-inducible “Cre” transgenic line, has also a slight effect on the baseline spontaneous AP firing frequency (Gao et al., 2013). In line with this, it was found that even a small NCX fraction is able to generate sufficient inward current to provide appropriate depolarization current which is able to maintain normal SAN cell activity (Groenke et al., 2013). Our and these previous results highlight the possibility that a functional coupling between I_f_ and NCX represents a potency to compensate for the NCX inhibition-induced reduction of the pacing frequency.

In accordance with this theory our and previous results indicate a relatively moderate effect of IVA on the CL when it is administrated at 3 µM (**Figure 4A**) or at 10 µM (Yaniv et al., 2012). At the same time, caesium was unable to stop SAN beating even though it has a large effect on I_f_ (Noma et al., 1983). These inconsistent results can be explained by the voltage-dependent block of IVA or caesium (DiFrancesco, 1995), or by proposing an insulator function of the I_f_ to protect the SAN cells from the strong negative electrical sink of the connected atrial tissue (Morad and Zhang, 2017). However, a functional coupling between I_f_ and NCX (Bois et al., 1996; Lakatta et al., 2010; Yaniv et al., 2013a) providing redundant pacemaking systems could also explain—or at least contribute to—the observed results. This phenomenon, which could be very similar to the repolarization reserve (Biliczki et al., 2002; Herrmann et al., 2007; Lengyel et al., 2007; Nagy et al., 2009), may be also able to reduce the effects of the individual inhibition of NCX or I_f_ explaining the relatively small extent of IVA or NCX effects.

Indeed, we found that the effect of 1 µM ORM gradually increased as the rate of I_f_ block was enhanced (**Figure 4**). In line with this, the Yaniv model provided similar but steeper tendency, when we represented the 0.5, 1, and 3 µM IVA doses by 20, 60, and 80% I_f_ block based on previous results (Bois et al., 1996). While 10 µM IVA was not used experimentally due to selectivity problems, 85% I_f_ inhibition could be computed in the Yaniv model. The detailed modeling results are depicted in **Figures 4E, F**. Consistent with experimental results, the effect of 41% NCX inhibition on CL is increased as I_f_ inhibition becomes stronger. However, the modeling predicts a much steeper increase in the CL in the presence of enhancing I_f_ block. The underlying mechanism of this discrepancy could be the markedly higher Ca^2+^ increase measured during experiments which could limit the bradycardic effect of NCX inhibition. A previous study reported a decreased SR Ca^2+^ content after I_f_ inhibition by IVA (Yaniv et al., 2013a) demonstrating an indirect suppression of NCX during I_f_ inhibition. Our Ca^2+^ measurements (**Figure 4C**) indicate unchanged Ca^2+^ release after application of IVA, which may indicate that the underlying mechanism of increased ORM effect may be rather related with the increased sensitivity of DD when it is already inhibited by the I_f_ block.

### I_Kr_ Inhibition Mediated Bradycardia Does Not Alter the Effect of Na^+^/Ca^2+^ Exchanger Inhibition

It is possible to decelerate spontaneous frequency without major direct influence on I_f_ or NCX. The SAN rate was reduced by 100% I_Kr_ block (**Figure 5**) in which the developed decrease in the firing rate was mainly achieved by APD prolongation without or minimal change in diastolic interval—instead of I_f_ block, which markedly increases the diastolic interval without effect on APD. This also means that despite the bradycardia, I_f_ is intact during these experiments. In line with this, NCX inhibition provided a similar effect to the one which was experienced when NCX was inhibited individually in **Figure 2**. This observation could be explained by an I_f_ dependent compensation of NCX reduction. At the same time it also means that the mechanism of the bradycardia is important regarding the effect of NCX inhibition. It seems possible that I_f_ mediated bradycardia and concomitant increase in diastolic interval may be important in the I_f_-NCX interaction.

### Suppression of Ca^2+^_i_ Augments the Effect of Funny Current Inhibition

Assuming that a mutual interaction between I_f_ and NCX exists, this crosstalk should work in the opposite direction as well, i.e., a disturbance in the Ca^2+^ cycling should affect I_f_. The suppression of the Ca^2+^ handling by the subsequent application of ryanodine and ORM together caused ≈20% increase in the CL in line with previous results (Bucchi et al., 2003). Under this condition, the effect of 3 µM IVA was considerably larger compared with normal settings (≈45% *vs.* 20%, see **Figure 6C**). In line with this, we found similar augmentation of IVA effect (21% *vs.* 51%) when Ca^2+^ handling was suppressed by low extracellular Ca^2+^ (**Figure 7**). Experimental as well as modeling simulations suppose that the Ca^2+^ handling and thus the NCX current suppression decreases the flux of the depolarizing NCX current, increases the length of DD, thus, the suppressed net current underlying the DD provides improved effect for I_f_ inhibition.

### Funny Current-Na^+^/Ca^2+^ Exchanger Coupling Controls Cycle Length Variability

Previous studies (Yaniv et al., 2013a; Yaniv et al., 2015) reported that the I_f_-NCX coupling not only controls the current CL but it may have a crucial role in maintaining the normal rhythm of the SAN. Therefore, the increase of the CL variability could be a further indicator of the integrity of I_f_-NCX axis appearing after a considerable CL increase reporting significant I_f_-NCX uncoupling. Our results support this assumption indicating that after individual inhibition of I_f_ or NCX, not only the excessive CL increase is restricted but the SAN rhythm is also maintained. However, when both of I_f_ and NCX are suppressed, besides the marked CL increase, a perturbation in the rhythm also appeared indicating the exhausted capacity of the I_f_-NCX to depolarize the membrane during the DD (**Figure 8**). Since we could not reach complete inhibition of I_f_ and NCX in our experiments, we cannot estimate precisely the relative importance of these currents in the normal SAN rhythm. However, it seems possible that these currents contribute in the “depolarization reserve” (Herrmann et al., 2007) not only to the control of the current CL but also to the maintenance of the normal pacing rhythm as a consequence of the strong depolarizing of the I_f_-NCX crosstalk.

### Proposed Mechanism

We suggest that the observed NCX-I_f_ interplay is the consequence of the increased susceptibility of DD to any intervention when the DD was previously inhibited by another compound (Rocchetti et al., 2000; Zaza and Lombardi, 2001; Monfredi et al., 2014). This means that the bradycardic effect of NCX inhibition is larger when I_f_ was previously inhibited (independently from the Ca^2+^ handling). *Vice versa*, when the NCX was previously suppressed (as a consequence of reduced Ca^2+^ release) the decreased DD current density is more sensitive to changes, which increases the bradycardic effect of IVA.

## Conclusion

In the present study, we provide direct pharmacological evidence regarding the role of NCX in pacemaker mechanism by its selective inhibition with the novel, highly selective compound ORM-10962. We found that individual inhibitions of NCX or I_f_ cause only moderate bradycardia and rhythm disturbance. However, combined suppression of these currents acted synergistically and supports the hypothesis of mutual crosstalk between NCX and I_f_ in SAN even in multicellular tissue having important functional consequences. This means that individual inhibition of DD currents may have moderated effect on CL and variability under normal conditions because the underlying currents may be able to compensate each other. This important crosstalk may provide a considerable safety margin for SAN pacemaking.

## Study Limitations

Our study has three important limitations. 1) The action potentials measured in our experiments do not represent the characteristics of the core SAN cells. These cells are much more “follower” cells, having AP waveforms largely influenced by the cell-to-cell coupling. 2) The applied inhibitors (IVA, ORM, RYA) are not able to cause complete block of ion channels in the applied concentrations. Therefore, the described phenomena indicate only partial effects and not able to estimate the absolute contribution of NCX during the DD. 3) Since our aim was to explore ion current cooperation, our results represent ion channel function independent from the autonomic nervous system. The activation of the sympathetic or parasympathetic nervous system—or modulation of the β_1_/M_2_ receptors—intimately changes the cAMP, PKA, CaMKII levels which have effects on the DD currents, therefore the discussed I_f_-NCX coupling cannot be directly extrapolated to *in vivo* systems. The ion current crosstalk characterization during intact autonomic control requires further experiments.

## Data Availability Statement

All datasets generated for this study are included in the article/supplementary material.

## Ethics Statement

The animal study was reviewed and approved by Munkahelyi Állatkísérleti Bizottság (MÁB).

## Author Contributions

ZK performed ion current measurements and data analysis. NT performed fluorescent optical measurements, action potential measurements and data analysis. JS performed ion current measurements and data analysis. AL performed the computational modeling and data analysis, contributed to conception of the study as well as manuscript preparation and funding for the computational study. PG performed ion current measurements, GB and JP performed action potential measurements. NJ organized the database and ensured the financial support of the study. JL and PP contributed to the development of ORM-10962. JGYP contributed to manuscript preparation, AV and NN ensured the financial support of the study, contributed conception and design of the study, data analysis and visualization, and manuscript preparation. All authors contributed to manuscript revision, read and approved the submitted version.

## Funding

This work was supported by grants from the National Research Development and Innovation Office (NKFIH PD-125402 (for NN), FK-129117 (for NN), GINOP-2.3.2-15-2016-00006, the LIVE LONGER EFOP-3.6.2-16-2017-00006 project, the János Bolyai Research Scholarship of the Hungarian Academy of Sciences (for NN), the UNKP-18-4-SZTE-76 New National Excellence Program of the Ministry for Innovation and Technology (for NN), the EFOP 3.6.3 VEKOP-16-2017-00009 (for NT), the Hungarian Academy of Sciences and by the Orion Pharma (ORM-10962).

## Conflict of Interest

PP and JL are employed by Orion Pharma, which has been involved in the development of ORM-10962.

The remaining authors declare that the research was conducted in the absence of any commercial or financial relationships that could be construed as a potential conflict of interest.

## Abbreviations

AP, action potential; APD, action potential duration; CL, cycle length; CLV, cycle length variability; DD, diastolic depolarization; DI, diastolic interval; DOF, dofetilide; NCX, sodium-calcium exchanger; I_f_, funny-current; IVA, ivabradine; ORM, ORM-10962; RYA, ryanodine; SAN, sinoatrial-node.
